# How does the strength of selection influence genetic correlations?

**DOI:** 10.1002/evl3.201

**Published:** 2020-11-03

**Authors:** Stéphane Chantepie, Luis‐Miguel Chevin

**Affiliations:** ^1^ Centre d'Ecologie et des Sciences de la Conservation (CESCO), Muséum national d'Histoire naturelle, Centre National de la Recherche Scientifique Sorbonne Université Paris France; ^2^ Centre d'Ecologie Fonctionnelle et Evolutive (CEFE) University of Montpellier, CNRS, University of Paul Valéry Montpellier 3, EPHE, IRD France

**Keywords:** G matrix, genetic drift, genetic correlation, stabilizing selection, selection‐mutation‐drift equilibrium

## Abstract

Genetic correlations between traits can strongly impact evolutionary responses to selection, and may thus impose constraints on adaptation. Theoretical and empirical work has made it clear that without strong linkage and with random mating, genetic correlations at evolutionary equilibrium result from an interplay of correlated pleiotropic effects of mutations, and correlational selection favoring combinations of trait values. However, it is not entirely clear how change in the overall strength of stabilizing selection across traits (breadth of the fitness peak, given its shape) influences this compromise between mutation and selection effects on genetic correlation. Here, we show that the answer to this question crucially depends on the intensity of genetic drift. In large, effectively infinite populations, genetic correlations are unaffected by the strength of selection, regardless of whether the genetic architecture involves common small‐effect mutations (Gaussian regime), or rare large‐effect mutations (House‐of‐Cards regime). In contrast in finite populations, the strength of selection does affect genetic correlations, by shifting the balance from drift‐dominated to selection‐dominated evolutionary dynamics. The transition between these domains depends on mutation parameters to some extent, but with a similar dependence of genetic correlation on the strength of selection. Our results are particularly relevant for understanding how senescence shapes patterns of genetic correlations across ages, and genetic constraints on adaptation during colonization of novel habitats.

**Impact Summary**

When phenotypic traits are genetically correlated, the direction and rate of phenotypic evolution is altered relative to what would be predicted solely from their genetic variances, with potential impacts on adaptation to changing environments such as climate change. These genetic correlations between traits result from a combination of evolutionary forces, chiefly mutations jointly influencing multiple traits (pleiotropy), and natural/sexual selection favoring some combinations of trait values (correlational selection). However, it is unclear whether and how much the overall strength of selection influences the degree to which genetic correlations are shaped mostly by pleiotropic mutation, versus by correlational selection. Here, we show that the response to this question crucially depends on the population size, which determines the level of randomness in the evolutionary process resulting from genetic drift. In large populations with negligible genetic drift, the same equilibrium genetic correlation is reached, regardless of the strength of selection. In contrast in smaller populations, the strength of selection determines whether genetic correlations are mostly explained by mutation (under weak selection), or by a compromise between mutation and selection (under strong selection). Our theoretical results can be used to analyze and interpret empirical estimates of genetic correlations across ages, or in other situations where the strength of selection varies in a predictable way.

Adaptation is inherently a multidimensional problem. Organisms live in complex environments composed of multiple niche axes (Hutchinson 1957), which exert natural selection on phenotypes composed of multiple traits that get integrated during development (Fisher 1930). This complexity can limit the process of adaptive evolution. First, the mere fact that multiple traits are under selection can slow down adaptation, which has been described as the cost of complexity (Fisher 1930; Orr 2000). And second, genetic correlations between traits can constrain the response to selection for any of these traits, thereby limiting the ensuing increase in fitness by adaptive evolution (Lande 1979; Etterson and Shaw 2001; Hansen and Houle 2008; Agrawal and Stinchcombe 2009; Walsh and Blows 2009; Chevin 2013; Connallon and Hall 2018). The development of evolutionary quantitative genetics theory on these questions (Lande 1979) was soon followed by a related formalism for measuring selection on correlated characters (Lande 1979; Lande and Arnold 1983). This has fostered much interest in the past decades for measuring patterns of genetic correlations among traits, in order to quantify constraints on adaptation (reviewed in Agrawal and Stinchcombe 2009). Such constraints can also be interpreted geometrically (Walsh and Blows 2009), since genetic correlations can influence the major axis of genetic variation across multiple traits, orienting evolution along genetic lines of least resistance (Schluter 1996).

Beyond quantifying the consequences of genetic correlations on rates on adaptation, understanding what shapes constraints on adaptation ultimately requires investigating the factors that govern the evolution of the **G** matrix, which includes all the additive genetic variances of traits and covariances among traits (Lande 1979). This has been a topic of intense research, both theoretically and empirically. Theoretical work has made it clear that, in randomly mating populations, genetic correlations evolve in response to (i) correlated pleiotropic mutation effects on traits, and (ii) correlational selection favoring combinations of trait values between pairs of traits (Lande 1980; Turelli 1985). Random genetic drift may also play an important role (Jones et al. 2003), but this was mostly investigated through individual‐based simulations, and few analytical results exist to guide intuition in that respect. In addition, patterns of environmental change (Jones et al. 2004, 2012) and epistatic interactions among loci (Jones et al. 2014) can also influence the shape of the **G** matrix and evolution of genetic correlation, but we will not address them here.

On the empirical side, it was recently demonstrated that the genetic divergence of multiple traits across several *Drosophila* species is aligned with the major axis of both the **G** matrix of additive genetic variation within species, and the **M** matrix of mutation effects on these traits (Houle et al. 2017). Natural selection was not measured in that study, but another study on the same set of traits has demonstrated that their genetic correlations can evolve in response to experimental patterns of correlational selection (Bolstad et al. 2015).

Since genetic correlations result from a compromise between mutational correlations and correlational selection, we may wonder: How do they change as the strength of selection varies? And more generally, how does the overall shape of the **G** matrix change as a fitness peak becomes broader (thus causing weaker selection), or narrower (stronger selection), while keeping the same overall shape (as illustrated in Figure 1A)? This simple question has received surprisingly little attention, despite its general importance in evolutionary biology. In particular, it bears on our understanding of the evolution of senescence by mutation accumulation, whereby relaxed selection in later age classes allows for accumulation of more genetic variance of traits (Charlesworth and Hughes 1996). A multivariate extension of this argument might suggest that the **G** matrix becomes more similar to the mutation **M** matrix in older ages, because they undergo relaxed selection. However, the premises that underlie this argument have yet to be explored more thoroughly.

**Figure 1:**
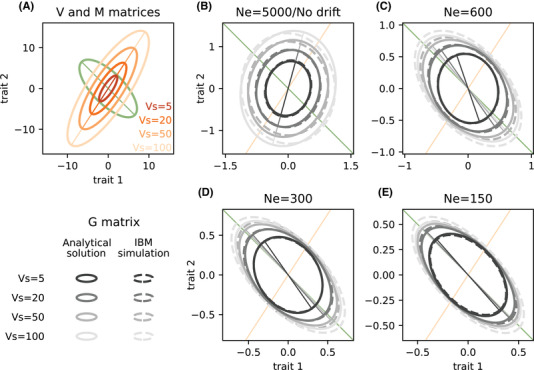
Influence of selection strength and genetic drift on the **G** matrix (Gaussian regime). (A) Orientation and shape of the mutation matrix M, and selection matrix V with variable selection strengths. First eigenvectors are also represented with colored lines. (B‐E) Shape and orientation of the G matrix as the width of the fitness peak $V_{s}$ varies. Solid ellipses (along with their first eigenvectors) represent the analytical predictions from equation (8) that neglects genetic drift in (B), or equation (9) that accounts for genetic drift in (C‐E). Dashed ellipses show the mean estimates from IBM simulations with $N_{e}=5000$ (B), 600 (C), 300 (D), and 150 (E). Standard data ellipses are represented, such that the half‐widths of their projections on axes *x* and *y* give the standard deviation of the corresponding traits (Friendly et al. 2013). The **M** ellipse (green) was magnified by a factor 5 × 10^4^ for graphical purpose. Parameters used: n=20, μ=0.01, $\rho_{m}=-0.7$, $\phi_{m}=1$, $V_{\alpha }=0.0025$ and $\rho_{s}=0.8$, $\phi_{s}=2$, and $V_{s}=5,20,50,100$.

Here, we investigate theoretically how the overall strength of selection influences evolution of genetic correlations, and the shape and orientation of the **G** matrix. Using analytical results and individual‐based simulations, we show that the relative importance of mutation versus selection in shaping the **G** matrix critically depends on random genetic drift.

## Methods

### MODEL

As in standard quantitative genetic models, we assume that the multivariate phenotype ***z*** can be partitioned into a breeding value ***x*** determined by the genotype, plus a residual component of variation ***e*** (often described as the environmental component), normally distributed with mean 0 and covariance matrix **E**. In each generation, mutations occur with probability μ at each allele of *n* diploid loci, such that the total mutation rate is 2nμ. Mutation increments the phenotypic value at the mutated allele by an effect that is unbiased (does not change the average breeding value), but can change the genetic (co)variances between traits. Specifically, we assume multivariate normally distributed mutation effects α, with mean 0 and the same covariance matrix **M** at each locus, which we parameterize (for two traits) as

(1) $$\begin{array}{cc} M & =V_{\alpha }M_{\rho }\\ M_{\rho } & =\left(\begin{array}{cc} 1 & \;\rho_{m}\sqrt{\phi_{m}}\\ \rho_{m}\sqrt{\phi_{m}} & \phi_{m} \end{array}\right). \end{array}$$

The parameter $V_{\alpha }$ is the variance of mutation effects on trait 1 (a scalar), $\phi_{m}$ is the ratio of mutational variances between traits 2 and 1, and $\rho_{m}$ is the mutational correlation. When $\phi_{m}=1$, the two traits have the same mutational variance, and $M_{\rho }$ is a mutational correlation matrix.

The multivariate phenotype is under stabilizing selection toward an optimum phenotype θ, which we assume constant for simplicity. This is modeled as classically by letting the fitness of individuals with multivariate phenotype ***z*** (relative to the fitness of the optimum phenotype) be

(2) $$W\left(z\right)=\exp\left(-\frac{{\left(z-\theta \right)}^{T}\Omega^{-1}\left(z-\theta \right)}{2}\right)$$

where the matrix Ω determines the breadth and orientation of the fitness peak. Averaging over the distribution of the residual phenotypic component ***e***, the fitness function on breeding values ***x***, which determines evolution of the **G** matrix, is

(3) $$\tilde{W}\left(x\right)\propto \exp\left(-\frac{{\left(x-\theta \right)}^{T}V^{-1}\left(x-\theta \right)}{2}\right)$$

where V=Ω+E is the stabilizing selection matrix, which can be written similarly to **M** as

(4) $$\begin{array}{cc} V & =V_{s}V_{\rho }\\ V_{\rho } & =\left(\begin{array}{cc} 1 & \;\rho_{s}\sqrt{\phi_{s}}\\ \rho_{s}\sqrt{\phi_{s}} & \phi_{s} \end{array}\right). \end{array}$$

The scalar $V_{s}$ determines the width of the fitness peak on breeding values, and is inversely proportional to the strength of stabilizing selection, while $\phi_{s}$ controls the ratio of strengths of selection between the two traits. The selective correlation $\rho_{s}$ determines what genetic correlation is favored by natural selection (see below). Figure 1A illustrates how these parameters translate into the shapes of the mutation and selection matrix.

### INDIVIDUAL‐BASED SIMULATIONS

We tested the accuracy of the expected G matrix and genetic correlation at mutation‐selection(‐drift) equilibrium using individual‐based, genetically explicit simulations. The traits were determined by n=20 unlinked diploid loci, with alleles assumed to be fully pleitropic (i.e., affecting all the phenotypic traits under selection). We simulated populations of hermaphroditic, sexually reproducing individuals with non‐overlapping generations. The life cycle included the following three steps:

1. **Assigning phenotype and fitness**. For each individual, its phenotype at each trait was computed by summing breeding values across all loci and alleles. This amounts to assuming no dominance and no epistasis at the phenotypic level (although they may exist at the fitness level), as also assumed in our analytical treatment. A residual component of variation was then added for each trait, with mean 0, variance 1, and no covariance between traits (that is, **E** was the identity matrix). The expected fitness of each individual was then computed based on its phenotypic value and the stabilizing selection matrix Ω, as defined by equation (2). The fitness optimum was arbitrarily set to zero.
2. **Selection and reproduction**. Two adults were drawn randomly with a probability proportional to their fitness, and mated to produce exactly one offspring. Selfing was not allowed, but beside this being involved in a reproductive event did not change the probability to mate again. The sequence was repeated N times, to yield parents for the N individuals of the next generation.
3. **Offspring production**. The genotype of each individual offspring was produced by drawing one random allele from each parent at each locus, thus modelling fully unlinked loci. The probability that a mutation occurred at each allele of each locus was μ. Mutation had additive effects on the traits, modifying the phenotypic value of the mutated allele by an amount drawn from a multivariate normal distribution, with mean zero (unbiased mutation) and mutation variance‐covariance matrix **M**.

This life cycle, developed by Revell (2007), ensures that the population size N is constant and equal to the effective population size $N_{e}$ in the absence of selection. However, something that has been largely overlooked in previous studies on this topic (e.g., Lande 1976, 1979; Burger et al. 1989), but becomes important under strong selection and low population size, is that selection on non‐heritable phenotypic variation increases the intensity of genetic drift on heritable phenotypic variation. This occurs because residual, non‐heritable phenotypic variation, with covariance matrix **E** (usually denoted as $V_{e}$ for single traits) causes variance in relative fitness among parents with a given breeding value x, thus increasing the amount of genetic drift, that is, random changes in the distribution of breeding values. As we show in online Appendix A, when all breeding values are close to the optimum (genetic variances small relative to the ”variance” of the fitness landscape), environmental variation reduces the effective population size to

(5) $$N_{e}=N\sqrt{\det[I-{\left({\left(\Omega +E\right)}^{-1}E\right)}^{2}]}$$

where N is the size of the parental population, possibly corrected for other factors affecting $N_{e}$ (such as uneven sex‐ratios; here, we assume a Wright‐Fisher population). Here, to account for the reduction in effective population size caused by selection on the residual component of variation, for a given required value of $N_{e}$ we used $N=N_{e}/\sqrt{\det[I-{\left({\left(\Omega +E\right)}^{-1}E\right)}^{2}]}$ as the population size in the simulations, to ensure that $N_{e}$ remains constant as the strength of selection changes.

Since all our formulas depended on the matrix V of selection on breeding values (eq. (3)), rather than the matrix Ω for selection on the expressed phenotype (eq. (2)), we parameterized simulations in terms of V, and then transformed them to Ω before starting the simulation, using Ω=V−E (as per eq. (3)), where E=I under our assumption of uncorrelated environmental effects with variance 1.

Individual‐based simulations were all run over 100,000 generations. To ensure that the expected genetic covariance matrix $\bar{G}$ was estimated after a pseudo‐equilibrium is reached (i.e., at stationarity), only the 70,000 last iterations from the chain were used to estimate the mean.

## Results

### WITHOUT DRIFT, GENETIC CORRELATIONS ARE UNCHANGED BY SELECTION STRENGTH

We will develop our argument about two traits for simplicity, but a similar reasoning applies for a larger number of traits. Using similar assumptions as here, Zhang and Hill (2003) showed that in an infinite population, and in the limit of rare mutations of large effect (so‐called House‐of‐Cards regime, HoC below; Turelli 1984, 1985; Bulmer 1989; Bürger 2000; Johnson and Barton 2005), the genetic correlation $\rho_{G}$ between two traits at mutation–selection balance with weakly linked loci is

(6) $$\rho_{G}=\frac{\rho_{s}\sqrt{1-\rho_{m}^{2}}+\rho_{m}\sqrt{1-\rho_{s}^{2}}}{\sqrt{2-\left(\rho_{m}^{2}+\rho_{s}^{2}\right)+\sqrt{\left(1-\rho_{s}^{2}\right)\left(1-\rho_{m}^{2}\right)}\left(\phi +\frac{1}{\phi }\right)}}$$

where $\phi =\sqrt{\frac{\phi_{m}}{\phi_{s}}}$. Remarkably, this shows that genetic correlations at mutation–selection balance depend neither on the absolute strength of stabilizing selection $V_{s}^{-1}$ nor on the magnitude of mutational variance $V_{\alpha }$, but instead on the ratio of strengths of stabilizing selection between the two traits, times the ratio of their mutation variances (summarized by the compound parameter ϕ). This means that narrowing the fitness peak, thereby increasing the strength of stabilizing selection on all traits, does not tilt the balance of genetic correlations $\rho_{G}$ toward selective correlations $\rho_{s}$ and away from mutational correlations $\rho_{m}$, as long as the overall shape of the mutation and selection matrices do not change. The same is true of increasing the mutational variance and covariances of all traits by the same factor. In the special case where ϕ=1, such that the the ratio of strengths of stabilizing selection on the two traits equals the ratio of their mutational variances (**M** and **V** have the same shape), equation (6) further simplifies as

(7) $$\rho_{G,\phi =1}=\frac{\rho_{s}\sqrt{1-\rho_{m}^{2}}+\rho_{m}\sqrt{1-\rho_{s}^{2}}}{\sqrt{1-\rho_{m}^{2}}+\sqrt{1-\rho_{s}^{2}}}$$

which only depends on mutational and selective correlations.

This result was obtained under the HoC regime, which is known to have different properties from a regime of common mutations of weak effect, known as the Gaussian regime (Kimura 1965; Lande 1976; Bulmer 1989; Bürger 2000; Johnson and Barton 2005). But in fact, genetic correlations also do not depend on the strength of selection under the Gaussian regime. To see this, we rewrite the equilibrium for the G matrix derived by Lande (1980) under the Gaussian regime, replacing V and M with their expressions in equations (1) and (4), to get

(8) $$G=2n\sqrt{\mu V_{\alpha }V_{s}}\;V_{\rho }^{\frac{1}{2}}{\left[V_{\rho }^{-\frac{1}{2}}M_{\rho }V_{\rho }^{-\frac{1}{2}}\right]}^{\frac{1}{2}}V_{\rho }^{\frac{1}{2}}.$$

The first scalar term is the same as for the genetic variance of a single trait at mutation‐selection balance in this regime (Kimura 1965; Lande 1976). It shows that the genetic variances and covariances of all traits, and hence the size of the **G** matrix, decrease as the strength of stabilizing selection increases (smaller $V_{s}$). The second term is a matrix that captures all the features of **G** matrix shape, including genetic correlations. Equation (8) shows that changing the overall strength of selection $V_{s}^{-1}$, or the scale of mutational variance $V_{m}$, only magnifies or shrinks the G matrix, but does not change genetic correlations in any way, nor any other aspect of G matrix shape.

**Figure 2:**
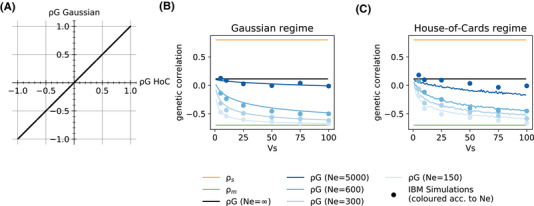
Influence of selection strength and genetic drift on genetic correlations under different mutation regimes. (A) The genetic correlation in an infinite population, at equilibrium between selection and abundant mutations of weak effects (Gaussian approximation, from eq. (8)) is plotted against its expectation under rare mutations of large effects (house‐of‐cards approximation, eq. (6)), for 500 random pairs of mutation M and selection V matrices. (B‐C) The genetic correlation is plotted against the width of the fitness peak $V_{s}$, for different effective sizes $N_{e}$. The parameters values in (B) are the same as in Figure 1, corresponding to the Gaussian mutation regime. In (C), the mutation parameters are instead $V_{\alpha }=0.05$ and μ=0.0002, corresponding to the House‐of‐cards regime. Blue points correspond to the genetic correlation simulated with individual‐based models (IBM). The black line represents the analytical expectation without drift (eq. (6)) in both cases. The blue lines represent the analytical prediction with drift (eq. (9)) in (B), and expectations over 10000 randomly drawn mutation effects α (from eq. (10)) in (C).

Figure 1 shows examples of **G** matrices under variable strengths of stabilizing selection (the fitness peak becomes broader, and selection becomes weaker, as $V_{s}$ increases, Fig. 1A), in the Gaussian regime. Continuous ellipses in Figure 1B represent the analytical prediction for **G** from equation (8), while dashed ellipses show results from genetically explicit individual‐based simulations (IBM) using the same parameters, but large finite population size, $N_{e}=5000$. The prediction that the strength of stabilizing selection does not affect the orientation of the G matrix in an infinite population is already close to holding in simulations with $N_{e}=5000$, indicating that such large populations behave as effectively infinite ones. The size of the **G** matrix increases, but its orientation and shape change little as the strength of selection decreases. This means that the strength of stabilizing selection affects the genetic variance of each trait (with less variance under stronger selection) and their covariance, but almost not their correlation. Figure 2B shows that the genetic correlation is indeed little influenced by the strength of selection in simulations with $N_{e}=5000$ (dark blue dots in Figure 2B), and remains close to the expected compromise between the mutational and selective correlations predicted by equation (6) (black line in Figure 2B), which does not depend on $V_{s}$.

Although the Gaussian and HoC regime have very different properties in terms of the maintenance of genetic variance for each trait (Turelli 1985; Bürger 2000), they strikingly lead to the same genetic correlation among traits in an infinite population. This was already suggested in numerical explorations by Turelli (1985), but we confirmed this here more extensively. In particular, when $\phi_{m}=\phi_{s}=1$, such that the mutation and selection matrices are both proportional to correlation matrices (with only 1 on the diagonal), then deriving the genetic correlation in the Gaussian case from the G matrix in equation (8) leads to equation (7), as in the HoC regime. In the more general case, an analytical formula also exists for the genetic correlation based on equation (8), but it is unwieldy. Instead of comparing Gaussian and HoC formulas for genetic correlation, we drew random matrices V and M from a Wishart distribution (with expectation I), a natural distribution for covariance matrices, which allows variance and covariance terms to vary randomly. We then computed the expected G matrix under the Gaussian regime (from eq. (8)), from which we extracted genetic correlations, which we then compared to equation (6). Figure 2A shows that the genetic correlation under the Gaussian regime, which assumes frequent mutations of small effects, is perfectly predicted by that under the House‐of‐Card regime, which instead assumes rare mutations of large effects. The blue dots in Figure 2C show genetic correlations for different values of the selection parameter $V_{s}$, estimated from individual‐based simulations with parameters that correspond to the HoC regime. These correlations are very similar to those in the Gaussian regime in Figure 2B, and close to their expectation in equation (6).

In short, genetic correlations at mutation‐selection balance do not change with the overall strength of stabilizing selection, and this conclusion holds generally across a broad range of mutation and selection parameters, spanning different evolutionary regimes. So should we then conclude that correlational selection always has the same influence on genetic correlations, and never becomes dominated by the influence of mutational correlations, even as the strength of selection becomes vanishingly small?

### DRIFT CONTROLS THE BALANCE BETWEEN MUTATION AND SELECTION'S EFFECTS ON GENETIC CORRELATIONS

In fact, genetic correlations may indeed become more similar to mutational correlations as the strength of selection decreases, but only in the presence of random genetic drift. In a population with finite effective size $N_{e}$, random genetic drift causes a reduction in heterozygosity, and thus in additive genetic variance, by a proportion $2N_{e}$ per generation. Accounting for this effect, we found that the expected G matrix at mutation‐selection‐drift equilibrium in the Gaussian regime is (Appendix B)

(9) $$\begin{array}{cc} \bar{G} & =2n\sqrt{\mu V_{\alpha }V_{s}}\;V_{\rho }^{\frac{1}{2}}\left[{\left(\kappa I^{2}+V_{\rho }^{-\frac{1}{2}}M_{\rho }V_{\rho }^{-\frac{1}{2}}\right)}^{\frac{1}{2}}-\sqrt{\kappa }I\right]V_{\rho }^{\frac{1}{2}}\\ \kappa & =\frac{V_{s}}{{\left(4N_{e}\right)}^{2}\mu V_{\alpha }} \end{array}$$

where $\bar{G}$ denotes an expectation over the stochastic evolutionary process (because of random genetic drift). As in equation (8), the first scalar term in equation (9) is the same as for the genetic variance of a single trait at mutation‐selection balance in this regime (Kimura 1965; Lande 1976), while the matrix product determines **G** matrix shape. Equation (9) shows that a single compound scalar parameter, $\kappa =V_{s}/\left[{\left(4N_{e}\right)}^{2}\mu V_{\alpha }\right]$, determines how the orientation and shape of the expected **G** matrix change under mutation, selection, and drift (since elements of $V_{\rho }$ and $M_{\rho }$ scale on the order 1 by construction). When $V_{s}\ll {\left(4N_{e}\right)}^{2}\mu V_{\alpha }$ (κ very small), the mutation rate and mean selection coefficient of new mutations are both large relative to the intensity of drift (proportional to $1/N_{e}$), so genetic correlations are mostly determined by mutation and selection, with little influence of genetic drift. In the limit κ→0, equation (9) tends to the mutation‐selection balance in equation (8). In contrast, drift dominates when $V_{s}\gg {\left(4N_{e}\right)}^{2}\mu V_{\alpha }$ (κ very large), and the expected G matrix then becomes increasingly similar to the mutation matrix M. This can be seen when comparing G matrices in panels B–E in Figure 1, as well as genetic correlations for different darknesses of blue in Figure 2B. Furthermore for a given $N_{e}$, the genetic correlation and orientation of the **G** matrix become more similar to those of mutation as the strength of selection decreases (increasing $V_{s}$, lighter ellipses in Fig. 1B–E, and rightmost values in Fig. 2B).

We have shown above that the type of mutation‐selection regime (HoC vs Gaussian) does not influence genetic correlations in an infinite population (Fig. 2A), but is it also the case in a finite population with substantial genetic drift? For the HoC regime, the expected G in an infinite population is proportional to the expectation of $\frac{\alpha \alpha^{t}}{\alpha^{t}V_{s}^{-1}\alpha }$ over the distribution of mutation effects α (Zhang and Hill 2003, eq. 2). In a finite population, accounting for the reduction in heterozygosity caused by both stabilizing selection and random genetic drift, this approximation becomes (adapted from Burger et al. 1989, ”stochastic house of cards” regime)

(10) $$\begin{array}{ccc} \bar{G} & = & 4N_{e}n\mu \;E\left[\frac{\alpha \alpha^{t}}{1+N_{e}\alpha^{t}V_{s}^{-1}\alpha }\right]\\ & = & 4n\mu V_{s}E\left[\frac{\alpha_{\rho }{\alpha_{\rho }}^{t}}{V_{s}/\left(N_{e}V_{\alpha }\right)+{\alpha_{\rho }}^{t}V_{\rho }^{-1}\alpha_{\rho }}\right], \end{array}$$

where E[] denotes an expectation over the distribution of mutation effects, and $\alpha_{\rho }=\alpha /\sqrt{V_{\alpha }}$ are scaled mutation effects, with covariance matrix $M_{\rho }$ as per equation (1). Analogously to equations (8) and (9), the first scalar term in equation (10) equals the equilibrium genetic variance for a single trait in the HoC regime, while the expectation includes all the parameters that determine G matrix shape. Since elements of $V_{\rho }$ and $M_{\rho }$ scale on the order 1 (and hence so do $\alpha^{t}V_{s}^{-1}\alpha$ and elements of $\alpha \alpha^{t}$), the scalar parameter $V_{s}/\left(N_{e}V_{\alpha }\right)$ alone determines whether the G matrix is more influenced by mutation, selection, or drift. When $V_{s}\ll N_{e}V_{\alpha }$, drift can be neglected and the G matrix has the same shape as in the HoC equilibrium; in particular, the genetic correlation between two traits is given by equation (6), and is thus the same as in the Gaussian regime. In contrast when $V_{s}\gg N_{e}V_{\alpha }$, drift dominate and the G matrix is proportional to M, with correlation $\rho_{m}$.

The stochastic house of cards approximation to the genetic correlation (eq. (10)) is somewhat less accurate at predicting results from individual‐based simulations than our stochastic Gaussian approximation in the corresponding regime (eq. (10), Fig. 2C, Fig. S5). However, it does capture a similar pattern, where the genetic correlation tends more rapidly toward the mutational correlational with decreasing strength of selection when the effective population size is smaller (Fig. 2C).

In summary, accounting for genetic drift in finite populations, the genetic correlation spans the same range in all mutation regimes, ranging from the mutational correlation $\rho_{m}$ when drift dominates, to the compromise between mutation and selection in equation (6) when selection dominates. The mutation regime (Gaussian vs HoC) only determines how the realized genetic correlation interpolates between these two limit cases. In particular, the selection strength $V_{s}^{-1}$ at which genetic correlations transition from being drift‐dominated to selection‐dominated is multiplied by ${\left(16N_{e}\mu \right)}^{-1}$ in the Gaussian regime relative to the HoC regime (eqs.(8) and (10)). When $16N_{e}\mu <1$, this means that stronger selection is required to overcome the influence of drift when mutations are abundant but with small effects (Gaussian regime) as compared to rare but with larger effects (and vice versa when $16N_{e}\mu >1$). But beyond these changes in the quantitative dependence on the strength of selection (which relate to previous findings for a single trait, Bürger 2000; Hermisson and Wagner 2004), the qualitative relationship between genetic correlations and the strength of selection given the effective population size remains the same across mutation regimes (Fig. 2B and C).

### SHAPE, ORIENTATION, AND CORRELATION

Changes in the relative importance of selection versus genetic drift can influence the shape of the **G** matrix (determined by its eigenvalues), its orientation (determined by its eigenvectors), or both. Any of these effects can translate into changes in genetic correlations, since the latter are only summaries of **G** matrice**s**, which depend on both variances and covariances (and on both eigenvectors and eigenvalues). In particular, genetic correlations may change despite little change in orientation, and with no rotation of the axes of the **G** matrix.

**Figure 3:**
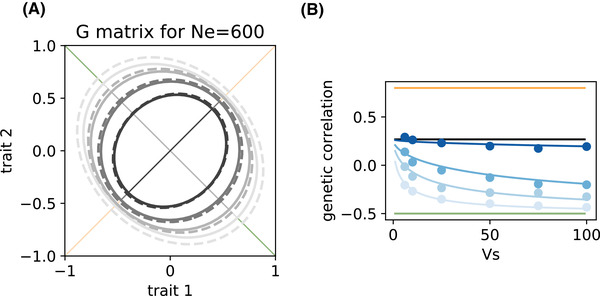
Influence of selection strength and genetic drift on genetic correlations in the special case where **V** and **M** have the same eigenvectors (Gaussian regime). (A) Shape and orientation of the **G** matrix as the width of the fitness peak $V_{s}$ varies. In this illustrative example where $N_{e}=600$, the first eigenvector of the **G** matrix (plain lines, from gray to black) is orientated either fully along the major axis for selection (eigenvector of **V**, orange line) or for mutation (eigenvector of **M**, green line), depending on the selection parameter $V_{s}$. (B) The genetic correlation is plotted against the width of the fitness peak $V_{s}$, for different effective sizes $N_{e}$ as in Figure 2. Note that, although axes of the **G** matrix do not rotate, the influence of selection strength and genetic drift on genetic correlations is comparable to the general case in Figure 2. Parameters are identical to Figure 1, except that $\phi_{s}=\phi_{m}=1$ such that **V** and **M** have the same eigenvectors, $\rho_{m}=-0.5$, and the strongest selection is for $V_{s}=6$ instead of 5. The shape of the **V** and **M** matrices, as well as **G** matrices for other values of $N_{e}$, are represented in figure S4.

This is illustrated in Fig. 3, which focuses on the special case where **V** and **M** have the same eigenvectors (which occurs for instance when $\phi_{s}=\phi_{m}=1$). When this holds, the eigenvectors of **G** are identical to those of **V** and **M**, as demonstrated in Appendix C. This means that the **G** matrix does not rotate when changing the relative importance of drift versus selection; all that changes are the amounts of variation (eigenvalues) along the different axes (eigenvectors). Nevertheless, the genetic correlation still changes according to equation (9) in this example. For instance, when one eigenvalue becomes dominant, the **G** matrix becomes increasingly elongated along one of the eigenvectors (Fig. 3A, Fig. S4), which translates into larger values of genetic correlations (Fig. 3B). In the more general case where **V** and **M** have different eigenvectors, then changing the relative importance of selection vs drift causes both elongation and rotation of **G** (changes in shape and orientation), but without necessarily causing larger changes in genetic correlations.

## Discussion and Conclusion

### SUMMARY AND INTERPRETATION OF RESULTS

To what extent are genetic correlations between traits shaped by natural selection, or imposed by mutation? This question, which in essence traces back to the debate between mutationists and selectionists in the early days of genetics (recently revived in the light of molecular evidence, Nei 2013), has received considerable attention from evolutionary biologists. Evolutionary quantitative genetic theory has made it clear that, under random mating and when linkage disequilibrium can be neglected, phenotypic (co)variances arise from an equilibrium between mutation and stabilizing selection, and genetic correlations are a compromise between the correlation of pleitropic mutation effects on traits, and correlational selection favoring combinations of traits (Lande 1980; Lande and Arnold 1983; Turelli 1985; Jones et al. 2003). The latter can be related to the orientation and elongation of the fitness landscape relating the traits to fitness (as illustrated in Fig. 1A). However beyond this shape of the fitness landscape, how does the overall strength of selection (size of the ellipses in Fig. 1A) influence genetic correlations between traits? As selection becomes weaker, the fitness peak becomes flatter, with a broader range of phenotypes having equivalent fitness, so does that reduce the influence of selection on genetic correlations? Perhaps surprisingly, the answer is no in an effectively infinite population, which in our simulations was already close to holding for a moderate effective size of $N_{e}=5000$. Strikingly, the same compromise between mutation and selection effects on genetic correlations holds regardless of the strength of selection, and regardless of whether genetic (co)variances are caused by common mutations of small effect (Gaussian regime, Kimura 1965; Lande 1980), or rare mutations of large effect (House‐of‐cards regime, Turelli 1984, 1985).

However, the strength of selection starts to matter as the effective populations size $N_{e}$ becomes smaller, and random genetic drift plays a larger role. The reason is that for a given $N_{e}$, the strength of selection shifts the balance between drift‐dominated and selection‐dominated evolutionary dynamics. Since genetic correlations equal mutational correlations $\rho_{m}$ in the former domain, but a compromise between mutation and selection (eq. (6)) in the latter, overall mutation has a stronger influence on genetic correlations than selection (Fig. 2). Previous analyses of **G** matrix evolution under mutation and correlational selection in finite populations has mostly focused on the effect of drift on the stability of the **G** matrix over evolutionary time (Jones et al. 2003), and largely overlooked the influence of drift on the expected **G**. In fact, this influence can be substantial, as shown here; in particular, it determines how the strength of selection affects genetic correlations.

Genetic correlations are often described as a constraint on adaptation (Etterson and Shaw 2001; Agrawal and Stinchcombe 2009; Connallon and Hall 2018), but this need not be true, depending on how the orientation of the **G** matrix relates to that of directional or fluctuating selection in a changing environment (Gomulkiewicz and Houle 2009; Duputié et al. 2012; Chevin 2013). In a constant environment as assumed here, the extent to which genetic correlations constrain adaptation depends on how the **G** matrix aligns with the matrix of correlational selection, represented in Figure 1A. Our analytical and simulation results show that genetic correlations, and the overall **G** matrix shape, differ more from those favored by correlational selection at lower effective population sizes. Since **G** becomes more similar to the mutation matrix **M** in this case, this could be interpreted as a mutational constraint on evolution (Nei 2013). However, this alignment with mutation effects occurs because of a prevalence of genetic drift, which is in fact the main constraint on adaptation in this case, also causing temporal fluctuations in the mean phenotype (Lande 1979) and in the G matrix itself (Jones et al. 2003), and apparent fluctuating selection (Chevin and Haller 2014).

### LIMITATIONS AND POSSIBLE EXTENSIONS

Our analytical results for genetic correlations and the **G** matrix at mutation‐selection‐drift balance in the Gaussian regime (eq. (9)) are valid under frequent mutation (Kimura 1965; Lande 1980; Bürger 2000), and we accordingly used high mutation rates in the corresponding simulations. However, note that the nature of loci is not explicit in this model, but in any case they do not represent single nucleotides or even genes. Rather, they represent large stretches of effectively non‐recombining portions of the genome, which may modify the traits by mutation. Since free recombination is also assumed across these loci (consistent with most previous studies), the latter can even be thought of as small chromosomes, for which mutation rates of the order of $10^{-2}$ seem reasonable. In addition, we also present theoretical and simulation results at much lower mutation rates (House‐of‐Cards regime), which lead to similar findings.

We assumed universal pleiotropy, whereby all loci have the same distribution of mutation effects on all traits. An interesting extension may be to allow for modular mutation effects, or restricted pleiotropy, whereby each locus can only modify a subset of traits by mutations (Chevin et al. 2010; Chebib and Guillaume 2017), to investigate whether the mutation regime has a stronger effect on mutation correlations in these scenarios. In terms of selection, we considered a fitness peak with an optimum, in line with most theory on the topic, but genetic correlations can also be favored by other forms of selection, notably disruptive selection (Bolstad et al. 2015), or negative frequency dependence caused by individual interactions (Mullon and Lehmann 2019), which may lead to different dependencies of genetic correlations on the strength of selection and genetic drift.

We also neglected the influence of linkage disequilibrium on genetic correlations and the **G** matrix, in line with previous theory on the topic (e.g. Jones et al. 2003), where unlinked loci were also assumed. However, although phenotypic associations between unlinked loci are individually small, they can collectively have a substantial impact on genetic (co)variances (and correlations) as the number of loci becomes large and selection is strong (Online Appendix E and Walsh and Lynch 2018). Our simulations show that this causes the influence of the strength of selection on genetic correlations to be reduced as the number of loci increases (Fig. S7, Fig. S8).

### BROADER IMPLICATIONS

Our results lead to predictions about changes in genetic correlations along lifetime. Genetic correlations between traits may differ among ages, if the phenotypic values at different ages are partly controlled by different loci. This assumption, which underlies both the mutation‐accumulation theory of senescence applied to quantitative traits (Charlesworth and Hughes 1996) and the theory of evolution of growth trajectories (Kirkpatrick and Lofsvold 1992), implies that trait values at different ages can be considered as different traits (or 'character states'). When this necessary condition holds, changes in genetic correlations among ages depend on where different ages lie along the evolutionary equilibria delineated above for the **G** matrix. All mutational and selective parameters affecting these equilibria may possibly change with age, but most of these changes are not expected to be in any particular direction, so their effects should cancel out across studies. In contrast, we do expect the strength of selection on all traits to generally decline with increasing age, because older ages contribute less to total fitness (they have smaller reproductive values, Lande 1982; Charlesworth 1993). All things being equal, based on our theoretical predictions (Fig. 2), we thus expect genetic correlations to lean more towards mutational correlations in older ages, but mostly when the effective population size is small. In contrast in large populations, genetic correlations may change in any direction (or not change) along lifetime. This pattern can be investigated by measuring genetic correlations among primary traits (not direct components of fitness) across ages, for different species that differ in effective population sizes (as estimated by e.g. their molecular polymorphism level).

More broadly speaking, we expect mutational correlations to impose more constraints on evolutionary trajectories in situations where the population size has been reduced, such as bottlenecks during colonization of novel habitats. Since these situations are also likely to be associated with strong directional selection, this should represent a double challenge for colonizing species. Nevertheless, the extent to which mutational correlations *per se* impede responses to directional selection is unclear. Even when genetic correlations are largely shaped by correlational selection (rather than just by mutation), they may still constrain adaptation, if directional selection in a novel or changing environment does not align with the shape of the fitness peak (Chevin 2013). In any case, our clear delineation of when, and how much, the strength of selection influences genetic correlations, should provide guidelines for analyzing and interpreting genetic constraints on adaptation in the wild.

## AUTHOR CONTRIBUTIONS

SC and LMC conceived the study. SC derived analytical solutions for the Gaussian regime. LMC derived analytical solutions for the House‐of‐Cards regime and effective size. SC developed and performed individual‐based simulations. SC and LMC wrote the manuscript.

## DATA ARCHIVING

The C++ software developed and used to performgenetically explicit individual‐based simulations is available at https://doi.org/10.6084/m9.figshare.13125455.v1.

Associate Editor: S. Wright

## Supporting information


**Data S1**.
**Figure S1**: Influence of selection on environmental variation of phenotypes on the effective population size.
**Figure S2**: Influence of selection strength and genetic drift on genetic correlations under Gaussian mutation regimes, assuming $N_{e}=N$.
**Figure S3**: Influence of selection strength and genetic drift on the G matrix under Gaus‐sian regime, assuming $N_{e}=N$.
**Figure S4**: Influence of selection strength and genetic drift on the G matrix when matri‐ces V and M have the same eigenvectors.
**Figure S5**: Influence of selection strength and genetic drift on the G matrix (House‐of‐Cards regime).
**Figure S6**: Influence of the number of loci on hidden genetic variance.
**Figure S7**: Influence of the number of loci on genetic correlations at mutation‐selection‐drift equilibrium.
**Figure S8**: Influence of the number of loci on genetic correlations at mutation‐selection‐drift equilibrium (Gaussian regimes, permuted correlations).
