# Supplementary material for: How does the strength of selection influence genetic correlations?
Source: Evol Lett. 2020 Nov 3;4(6):468–78. doi: 10.1002/evl3.201 (PMC7719553; doi:10.1002/evl3.201)
Supplement: Supplementary file 1 — Data S1. Figure S1: Influence of selection on environmental variation of phenotypes on the effective population size. Figure S2: Influence of selection strength and genetic drift on genetic correlations under Gaussian mutation regimes, assuming Ne=N. Figure S3: Influence of selection strength and genetic drift on the G matrix under Gaus‐sian regime, assuming Ne=N. Figure S4: Influence of selection strength and genetic drift on the G matrix when matri‐ces V and M have the same eigenvectors. Figure S5: Influence of selection strength and genetic drift on the G matrix (House‐of‐Cards regime). Figure S6: Influence of the number of loci on hidden genetic variance. Figure S7: Influence of the number of loci on genetic correlations at mutation‐selection‐drift equilibrium. Figure S8: Influence of the number of loci on genetic correlations at mutation‐selection‐drift equilibrium (Gaussian regimes, permuted correlations). [file EVL3-4-468-s001.pdf]

Online appendix to Chantepie & Chevin, Evolution Letters

A Influence of selection on non-heritable phenotypic variation on genetic drift

Non-heritable phenotypic variation, often described as environmental variance (denoted as V_e in standard univariate quantitative genetics) can increase the intensity of drift for the genetic component of traits under selection. This occurs because environmental variance of a trait affecting fitness causes variance in reproductive success conditional on the genetic value of this trait, which reduces the effective population size and increases the intensity of drift.

Conditional on the genetic value $\mathbf{x}$ of the trait, each individual has a different phenotype determined by the continuously distributed (multivariate) environmental value $\mathbf{e}$, and the fitness of an individual i with environmental value $\mathbf{e}_i$ is $W_i = W(\mathbf{x}+\mathbf{e}_i)$. The probability that this individual is chosen to contribute any given gamete composing the offspring population is $p_i = \frac{W_i}{N\widetilde{W}(\mathbf{x})}$, where $\widetilde{W}(\mathbf{x})$ is the mean fitness of individuals with breeding value $\mathbf{x}$, integrated over the distribution of $\mathbf{e}$. If the population has constant size, there are $2N$ such gametes in the offspring population, so the numbers $(k_1, k_2, ..., k_i, ...k_N)$ of gametes contributed by each of the N parents are drawn from a multinomial distribution with size $2N$ and probability vector $\mathbf{p} = (p_1, p_2, ..., p_i, ...p_N)$. To derive the inbreeding effective size, which most directly relates to the reduction of genetic variance of a quantitative trait caused by drift, we need to find the probability P that two randomly chosen gametes in the offspring come from the same parent (following e.g. Crow and Kimura 1970, p345). For any given parent i leaving k_i offspring, this equals $\frac{k_i(k_i-1)}{2N(2N-1)}$, which is then summed over parents to yield

$$P = \frac{\sum_{i=1}^{N} k_i(k_i-1)}{2N(2N-1)}. \tag{S1}$$

The expected probability averaged over the sampling process is then

$$
\begin{aligned}
E(P) &= \frac{\sum_{i=1}^{N} E(k_i(k_i-1))}{2N(2N-1)} \\
&= \frac{\sum_{i=1}^{N} E(k_i^2-k_i)}{2N(2N-1)} \\
&= \frac{\sum_{i=1}^{N}(Var(k_i)+E(k_i)^2-E(k_i))}{2N(2N-1)} \\
&= \frac{\sum_{i=1}^{N}(2Np_i(1-p_i)+(2Np_i)^2-2Np_i)}{2N(2N-1)} \\
&= \frac{\sum_{i=1}^{N} 2N(2N-1)p_i^2}{2N(2N-1)} \\
&= \sum_{i=1}^{N} p_i^2 = \frac{\sum_{i=1}^{N} W_i^2}{N^2\widetilde{W}^2} = \frac{\widetilde{W^2}}{N\widetilde{W}^2}
\end{aligned}
\tag{S2}
$$

where tildes again denote averages over the distribution of environmental values in the population. Then the inbreeding effective population size is simply

$$
N_e = \frac{1}{E(P)} = N\frac{\widetilde{W}^2}{\widetilde{W^2}} = \frac{N}{1+CV^2(w)} \tag{S3}
$$

where CV denotes a coefficient of variation over the distribution of environmental values, and $w = W/\widetilde{W}$ is relative fitness conditional on breeding value. Note that the formula in equation (S3) (which appeared previously in e.g. Santiago and Caballero 1995) differs from more common ones based on the variance in reproductive success $Var(k)$ (e.g. Crow and Kimura 1970, p345-352), because we here focus on the variance in relative fitness w, which determines the *expected* (rather than realized) number of offspring with a given phenotype (as clarified by Robertson 1961). Note also that we do not allow self-fertilization in our simulations, which should increment N_e by approximately half an individual (Wright 1969, p.195), but we neglect this for simplicity.

With the fitness function in equation (2) the mean fitness of individuals with breeding value $\mathbf{x}$ is:

$$
\tilde{W}(\mathbf{x}) = \sqrt{\det((\mathbf{\Omega}+\mathbf{E})^{-1}\mathbf{\Omega})}\exp\left(-\frac{(\mathbf{x}-\boldsymbol{\theta})^T(\mathbf{\Omega}+\mathbf{E})^{-1}(\mathbf{x}-\boldsymbol{\theta})}{2}\right) \tag{S4}
$$

and the mean squared relative fitness (conditional on breeding value) is

$$
\frac{\widetilde{W^2}(\mathbf{x})}{\widetilde{W}^2(\mathbf{x})} = \frac{\sqrt{\det((\mathbf{\Omega}+2\mathbf{E})^{-1}\mathbf{\Omega})}}{\det((\mathbf{\Omega}+\mathbf{E})^{-1}\mathbf{\Omega})}\exp\left(-(\mathbf{x}-\boldsymbol{\theta})^T[(\mathbf{\Omega}+2\mathbf{E})^{-1}-(\mathbf{\Omega}+\mathbf{E})^{-1}](\mathbf{x}-\boldsymbol{\theta})\right) \tag{S5}
$$

which can be rearranged as

$$\frac{\widetilde{W^2}(\mathbf{x})}{\widetilde{W}^2(\mathbf{x})} = \frac{1}{\sqrt{\det[\mathbf{I} - ((\mathbf{\Omega} + \mathbf{E})^{-1}\mathbf{E})^2]}} \exp\left((\mathbf{x} - \boldsymbol{\theta})^T(\mathbf{\Omega} + 2\mathbf{E})^{-1}\mathbf{E}(\mathbf{\Omega} + \mathbf{E})^{-1}(\mathbf{x} - \boldsymbol{\theta})\right). \quad \text{(S6)}$$

In the simplest case where the mean breeding value is at the optimum and genetic variation is small (conditions provided below), then the exponential in equation (S6) equals 1, and we have

$$N_e = N\sqrt{\det[\mathbf{I} - ((\mathbf{\Omega} + \mathbf{E})^{-1}\mathbf{E})^2]}. \quad \text{(S7)}$$

The univariate version for a single trait is

$$N_e = N\sqrt{1 - \left(\frac{V_e}{Ve + \omega^2}\right)^2} \quad \text{(S8)}$$

where V_e is the environmental variance of the trait and ω the width of the fitness peak.

However, when the distribution of breeding values is sufficiently broad (large genetic variance for at least some of the traits), then the effect of environmental variance on genetic drift should be averaged over the distribution of breeding values. Integrating equation (S6) over the distribution of breeding values $\mathbf{x}$ with mean at the optimum, and taking the reciprocal, we get

$$N_e = N\sqrt{\det[((\mathbf{I} - (\mathbf{\Omega} + \mathbf{E})^{-1}\mathbf{E})^2(\mathbf{I} - 2\mathbf{\Phi}\mathbf{G})]} \quad \text{(S9)}$$

where $\mathbf{\Phi}$ is the matrix in the exponential of equation (S6) and $\mathbf{G}$ is the genetic covariance matrix. This shows that the influence of genetic variation on how environmental variation affects the effective population size can be neglected as long as $2\mathbf{\Phi}\mathbf{G}$ is small relative to $\mathbf{I}$, in which cases equation (S6) collapses to equation (S7). For a single trait, this condition can be shown to be equivalent to $2V_g \ll 3\omega^2$ (with V_g the additive genetic variance), that is, weak stabilizing selection on genetic variance.

To check the validity of equation (S7), we ran simple simulations with selection on non-heritable phenotypic variation, and drift at a neutral bi-allelic locus. We initiated the population by randomly drawing each gene copy of N diploid individuals with equal probability 1/2 between two alleles A and B. We then recorded the initial frequency p_0 of allele A (which may slightly differ from 1/2 because of random sampling), and the initial heterozygosity $H_0 = 2p_0(1 - p_0)$. We then iterated 10000 times the following process:

1. Randomly draw a multivariate residual component of variation $\mathbf{e}$ for each individual, with mean 0 and covariance matrix $\mathbf{E}$.

2. Compute the fitness of each individual using equation (2) with the optimum set at 0.

3. Sample N pairs of mating parents with repeat (allowing for selfing), with weights given by their fitnesses.

4. Randomly draw one allele from each parent (segregation), to create the diploid genotype of each individual in the offspring generation.

5. Compute the new frequency p_1 and heterozygosity $H_1 = 2p_1(1 - p_1)$ in the offspring generation.

From this we could compute both the variance effective population size

$$N_{e,V} = \frac{p_0(1 - p_0)}{2Var(p_1)} \tag{S10}$$

and the inbreeding effective population size

$$N_{e,I} = \frac{H_1}{2(H_0 - E(H_1))} \tag{S11}$$

where the expectations and variances are taken over the 10000 simulations (Crow and Kimura 1970). The comparison between these simulated results and the predictions from equation (S7) is shown in Figure S1.

In addition, we also ran multi-locus individual-based simulations as in the main text, except that we did not correct the population size N for the influence of selection on environmental variation, causing N_e to change with the strength of selection. Results are shown in Figures S2 and S3.

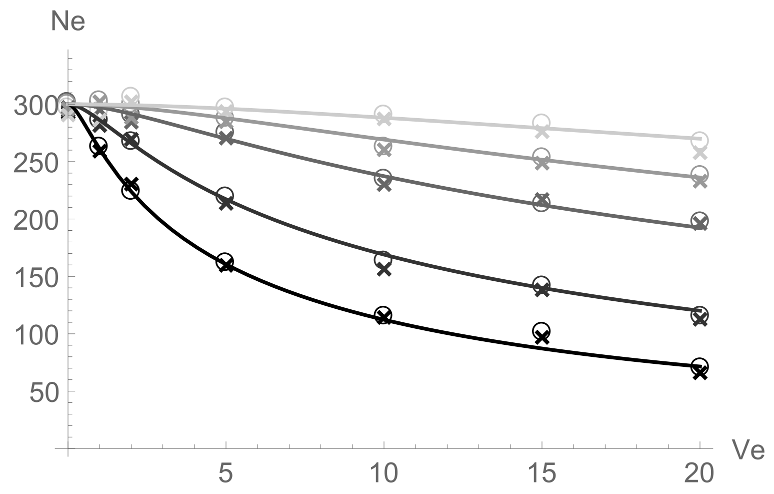

Figure S1: **Influence of selection on environmental variation of phenotypes on the effective population size.** Open circles show variance effective population size (eq. (S10)) and crosses show inbreeding effective population sizes (eq. (S11)), both computed from 10000 simulations of drift at a neutral bi-allelic locus, in a population of size $N = 300$. The lines show predictions from equation (S7). The selection matrix was $\mathbf{\Omega} = \omega^2 \mathbf{\Omega}_\rho$, with $\mathbf{\Omega}_\rho = \left(\begin{smallmatrix} 1 & 0.8\sqrt{2} \\ 0.8\sqrt{2} & 2 \end{smallmatrix}\right)$ and $\omega = 2, 3, 5, 7, 10$ from black to light gray. The environmental covariance matrix was $\mathbf{E} = V_e \mathbf{I}$, with $V_e = 0.001, 1, 2, 5, 10, 15, 20$.

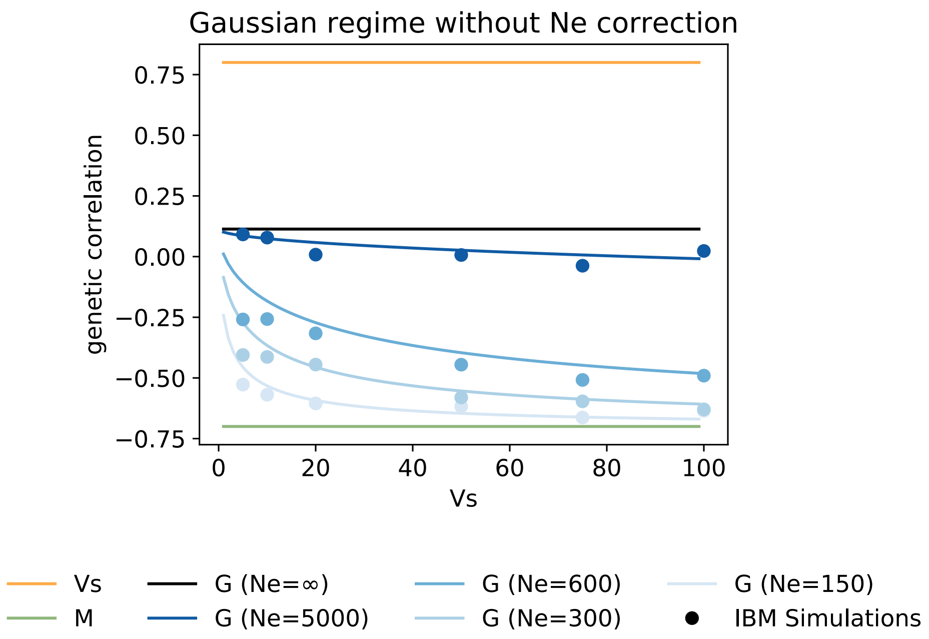

Figure S2: **Influence of selection strength and genetic drift on genetic correlations under Gaussian mutation regimes, assuming $N_e = N$.** The genetic correlation is plotted against the width of the fitness peak V_s, for different populations sizes N, uncorrected for the influence of environmental variation on N_e. The parameters values are the same as in Figure 1, corresponding to the Gaussian mutation regime. A detailed legend is provided in Figure 2.

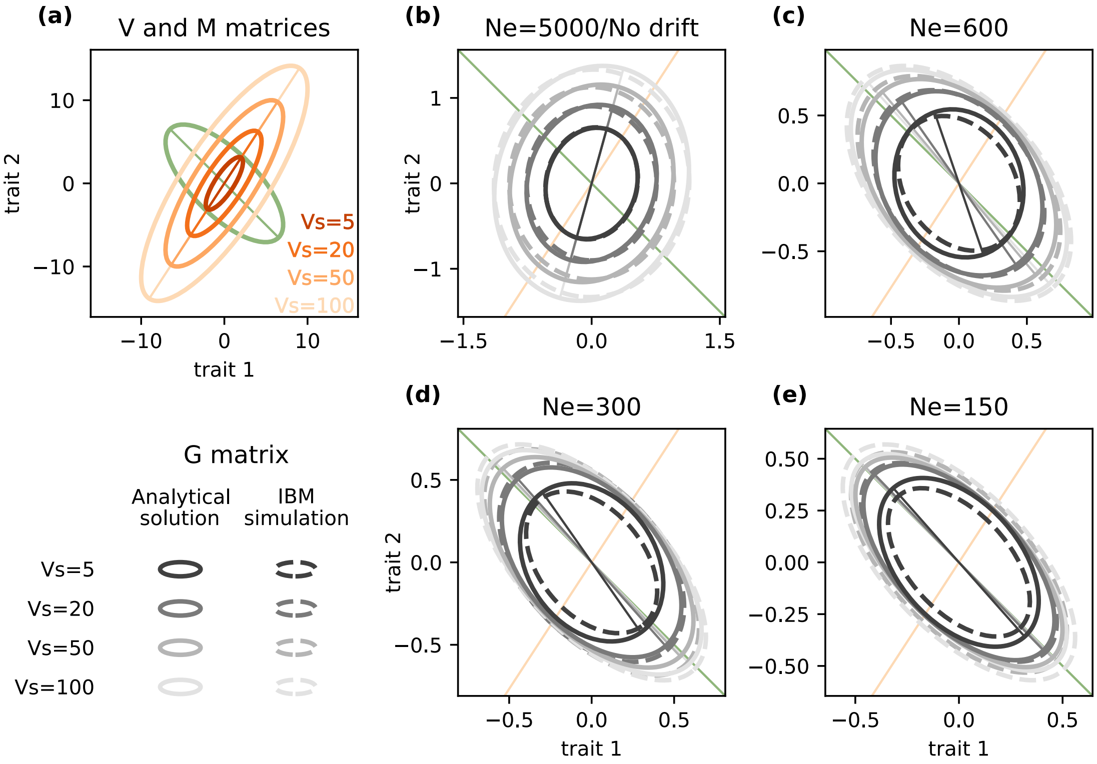

Figure S3: **Influence of selection strength and genetic drift on the G matrix under Gaussian regime, assuming $N_e = N$.**(a) Orientation and shape of **M** and **V** matrices for parameters values similar to Figure 1 (b-f) see Figure 1 for a detailed legend.

B Steps for solving the mutation-selection-drift equilibrium

By building on the Gaussian approximation of the continuum-of-allele model (Kimura 1965) and assuming infinite-sized population with non-overlapping generations, Lande (1980) derived a simple expression for genetic covariance matrix equilibrium ($\mathbf{G}$) selection-mutation balance. We here extend this result to allow for random genetic drift. At equilibrium, the production of genetic variance due to new polygenic mutations is balanced by the loss of genetic variance to due both stabilizing selection and random drift (Lande 1979, 1980). For a single haploid locus we obtain :

$$\mu\mathbf{M} = \overline{\mathbf{G}}\mathbf{V}^{-1}\overline{\mathbf{G}} + \frac{1}{2Ne}\overline{\mathbf{G}}$$

where $\overline{\mathbf{G}}$ denotes an expectation over the stochastic evolutionary process (because of random genetic drift).

Introducing $\mathbf{B} = \frac{1}{2Ne}\mathbf{I}$ and $\mathbf{U} = \mu\mathbf{M}$, this becomes

$$\mathbf{U} = \overline{\mathbf{G}}\mathbf{V}^{-1}\overline{\mathbf{G}} + \mathbf{B}\overline{\mathbf{G}}$$

$$\mathbf{V}^{-\frac{1}{2}}\mathbf{U}\mathbf{V}^{-\frac{1}{2}} = \mathbf{V}^{-\frac{1}{2}}\overline{\mathbf{G}}\mathbf{V}^{-\frac{1}{2}}\mathbf{V}^{-\frac{1}{2}}\overline{\mathbf{G}}\mathbf{V}^{-\frac{1}{2}} + \mathbf{V}^{-\frac{1}{2}}\mathbf{B}\overline{\mathbf{G}}\mathbf{V}^{-\frac{1}{2}}$$

By defining $\mathbf{V}^{-\frac{1}{2}}\overline{\mathbf{G}}\mathbf{V}^{-\frac{1}{2}} = \mathbf{X}$ and $\mathbf{V}^{-\frac{1}{2}}\mathbf{U}\mathbf{V}^{-\frac{1}{2}} = -\mathbf{C}$, we finally have to solve the quadratic matrix equation :

$$\mathbf{0} = \mathbf{X}^2 + \mathbf{B}\mathbf{X} + \mathbf{C}$$

A quadratic matrix equation can be solved explicitly if the following requirements are met: (i) $\mathbf{X}^2$ is preceded by an identity matrix, (ii) $\mathbf{B}$ commutes with $\mathbf{C}$, and (iii) $\mathbf{B}^2 - 4\mathbf{C}$ has a square root. The solution of the quadratic matrix equation is then :

$$\mathbf{X} = -\frac{1}{2}\mathbf{B} + \frac{1}{2}\left(\mathbf{B}^2 - 4\mathbf{C}\right)^{\frac{1}{2}}$$

In our case, an explicit solution for $\mathbf{X}$ exists. Indeed, $\mathbf{X}^2$ is preceded by an identity matrix, $\mathbf{B}$ is a diagonal matrix and then always commute with $\mathbf{C}$. Finally, as $\mathbf{B}^2$ and $\mathbf{C}$ are both positive semi-definite then $(\mathbf{B}^2 - 4\mathbf{C})^{1/2}$ will always have a solution. Recall that

$$\mathbf{V}^{-\frac{1}{2}}\overline{\mathbf{G}}\mathbf{V}^{-\frac{1}{2}} = \mathbf{X}$$

then

$$\overline{\mathbf{G}} = \mathbf{V}^{\frac{1}{2}}\mathbf{X}\mathbf{V}^{\frac{1}{2}}$$

$$\overline{\mathbf{G}} = \mathbf{V}^{\frac{1}{2}} \left[-\frac{1}{2}\mathbf{B} + \frac{1}{2}\left(\mathbf{B}^2 - 4\mathbf{C}\right)^{\frac{1}{2}} \right] \mathbf{V}^{\frac{1}{2}}$$

$$\overline{\mathbf{G}} = \mathbf{V}^{\frac{1}{2}} \left[-\frac{1}{4Ne}\mathbf{I} + \frac{1}{2}\left(\frac{1}{(2Ne)^2}\mathbf{I}^2 + 4\mathbf{V}^{-\frac{1}{2}}\mathbf{U}\mathbf{V}^{-\frac{1}{2}} \right)^{\frac{1}{2}} \right] \mathbf{V}^{\frac{1}{2}}$$

Finally by using notations (1) and (4), and summing over diploid loci (neglecting linkage disequilibrium), we obtain equation (9)

C Constraints on the orientation and shape of the G matrix in the special cases where V and M matrices have the same eigenvectors

C.1 Case without drift

Starting from equation (8)

$$\mathbf{G} = 2n\sqrt{\mu V_\alpha V_s} \quad \mathbf{V}_\rho^{\frac{1}{2}} \left[\mathbf{V}_\rho^{-\frac{1}{2}} \mathbf{M}_\rho \mathbf{V}_\rho^{-\frac{1}{2}}\right]^{\frac{1}{2}} \mathbf{V}_\rho^{\frac{1}{2}}$$

and assuming that both matrices $\mathbf{V}_\rho$ and $\mathbf{M}_\rho$ have the same eigenvectors $\mathbf{Q}$ but different eigenvalues, their spectral decompositions give $\mathbf{V}_\rho = \mathbf{Q}\mathbf{\Lambda}_s\mathbf{Q}^{-1}$ and $\mathbf{M}_\rho = \mathbf{Q}\mathbf{\Lambda}_m\mathbf{Q}^{-1}$ respectively, where $\mathbf{\Lambda}_w$ and $\mathbf{\Lambda}_m$ are diagonal matrices of eigenvalues. Then, equation (8) can be simplified to

$$\mathbf{G} = 2n\sqrt{\mu V_\alpha V_s} \quad \mathbf{Q}\left[\mathbf{\Lambda}_w \mathbf{\Lambda}_m\right]^{\frac{1}{2}} \mathbf{Q}^{-1}$$

This shows that $\mathbf{G}$ matrix has the same eigenvectors $\mathbf{Q}$ as the $\mathbf{V}$ and $\mathbf{M}$ matrices, and that its eigenvalues are the geometric means of eigenvalues of $\mathbf{V}$ and $\mathbf{M}$.

C.2 Case with drift

Starting from equation (9)

$$\overline{\mathbf{G}} = 2n\sqrt{\mu V_\alpha V_s} \quad \mathbf{V}_\rho^{\frac{1}{2}} \left[\left(\frac{V_s}{(4N_e)^2\mu V_\alpha}\mathbf{I}^2 + \mathbf{V}_\rho^{-\frac{1}{2}} \mathbf{M}_\rho \mathbf{V}_\rho^{-\frac{1}{2}}\right)^{\frac{1}{2}} - \sqrt{\frac{V_s}{(4N_e)^2\mu V_\alpha}}\mathbf{I}\right] \mathbf{V}_\rho^{\frac{1}{2}}$$

and performing a spectral decomposition of $\mathbf{V}_\rho$ and $\mathbf{M}_\rho$ as in the case without drift (see above), after simplification we obtain :

$$\overline{\mathbf{G}} = 2n\sqrt{\mu V_\alpha V_s} \quad \mathbf{Q}\left[\left[\frac{V_s}{(4N_e)^2\mu V_\alpha}\mathbf{\Lambda}_s^2 + \mathbf{\Lambda}_s\mathbf{\Lambda}_m\right]^{\frac{1}{2}} - \frac{V_s}{(4N_e)^2\mu V_\alpha}\mathbf{\Lambda}_s\right] \mathbf{Q}^{-1}$$

While the eigenvalues of $\mathbf{G}$ are given by the equation located between the highest level of brackets, the eigenvectors of $\mathbf{G}$ still equal $\mathbf{Q}$ the same as $\mathbf{V}$ and $\mathbf{M}$ matrices.

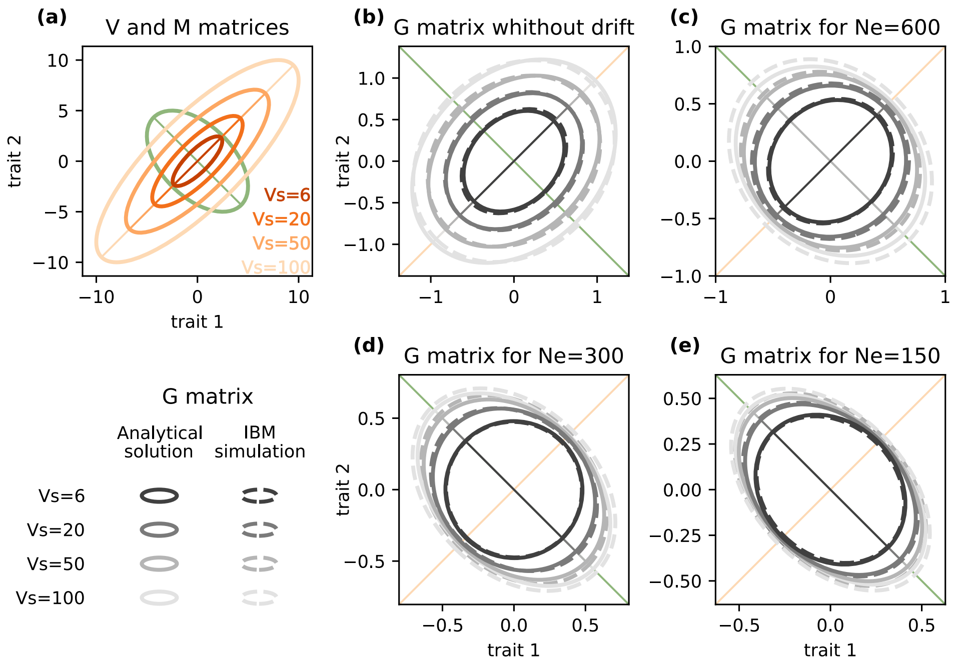

Figure S4: **Influence of selection strength and genetic drift on the G matrix when matrices V and M have the same eigenvectors.** (a) Orientation and shape of **M** and **V** matrices for respectively $\rho_m = -0.5$, $\phi_m = 1$, $V_\alpha = 0.05$, $\rho_s = 0.7$,$\phi_s = 1$, $V_\alpha = 6, 20, 50, 100$. (b-f) see Figure 1 for a detailed legend.

D G matrix in the House of cards regime

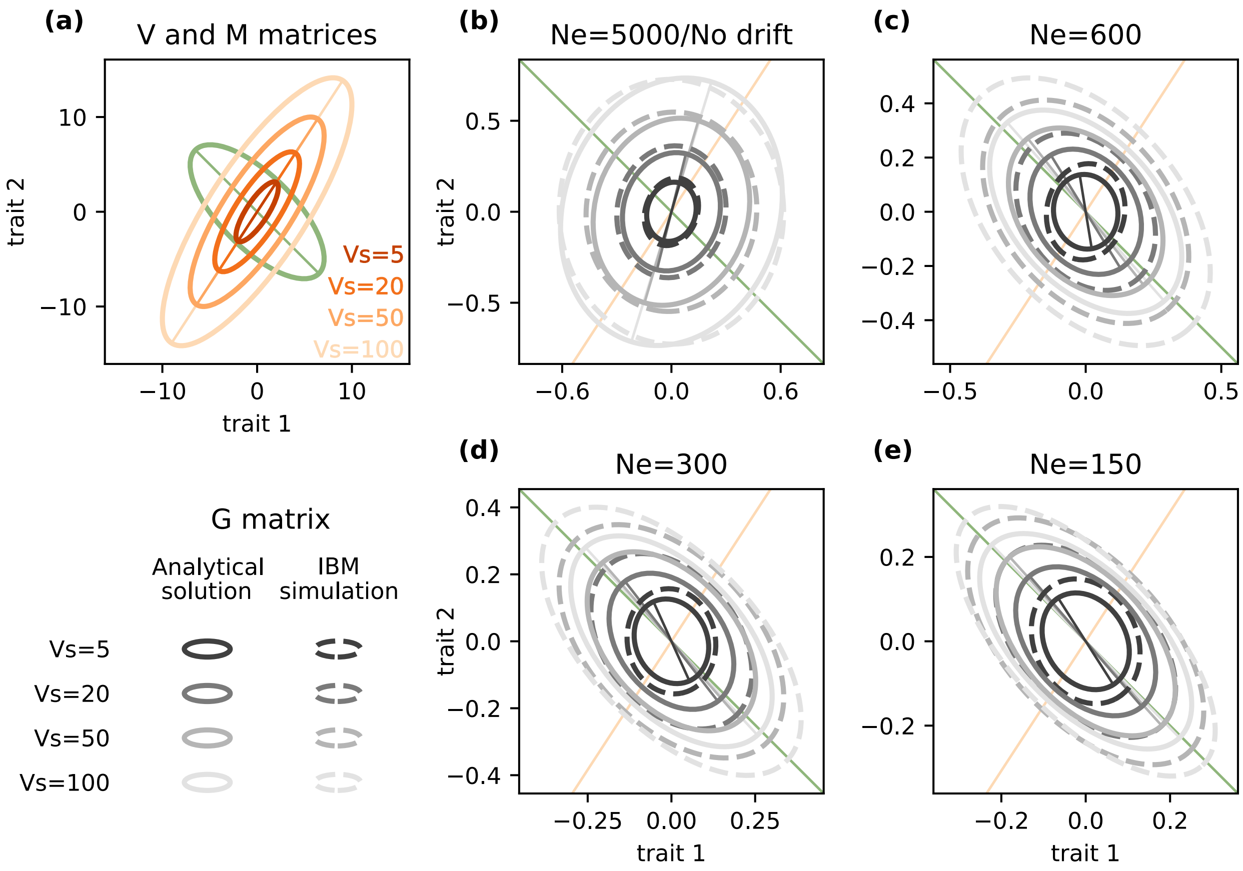

Figure S5: **Influence of selection strength and genetic drift on the G matrix (House-of-Cards regime).** (a) Orientation and shape of **M** and **V** matrices for respectively $\rho_m = -0.7$, $\phi_m = 1$, $V_\alpha = 0.05$ and $\rho_s = 0.8$, $\phi_s = 2$, $V_s = 5, 10, 50, 100$. First eigenvectors are represented colored lines. We set $n = 20$ and the $\mu = 0.0002$. (b-f) see Figure 1 for a detailed legend.

E Influence of the number of loci on genetic correlation

We have assumed in our analysis that linkage disequilibrium between loci does not contribute to genetic correlations between traits. As shown by Lande (1980), this assumption is generally reasonable for weak selection and unlinked loci as modeled here, and in previous theory on the topic (e.g. Jones *et al.* 2003). However under strong stabilizing selection, and as the number of loci becomes large, genetic covariation among loci can bias genetic correlations away from our analytical predictions above, even without linkage.

The true additive genetic covariance matrix is

$$\mathbf{G} = \sum_{i=1}^{n} \mathbf{G}_{ii} + 2\sum_{i=1}^{n-1}\sum_{j=i+1}^{n} \mathbf{G}_{ij} = \mathbf{G}_g + \mathbf{G}_{LD}, \tag{S12}$$

where $\mathbf{G}_{ii}$ is the additive genetic covariance matrix at locus i, and $\mathbf{G}_{ij}$ is the additive genetic cross-covariance matrix between loci i and j. The first sum is generally described as the genic covariance matrix $\mathbf{G}_g$ (Walsh and Lynch 2018, p.550), while the second sum $\mathbf{G}_{LD}$ reflects the influence of linkage disequilibrium between loci. For equivalent loci, we further have $\mathbf{G}_g = n\mathbf{G}_w$ and $\mathbf{G}_{LD} = n(n-1)\mathbf{G}_b$, where $\mathbf{G}_w$ and $\mathbf{G}_b$ are respectively the within-locus and between-locus additive genetic covariance matrices. Our analytical results in the main text have only considered the genic covariance matrix $\mathbf{G}_g$, in effect neglecting linkage disequilibrium, in line with most previous theory (Bulmer 1989; Burger *et al.* 1989; Jones *et al.* 2003). But in reality, stabilizing selection produces negative linkage disequilibrium between loci, causing $\mathbf{G}_{LD}$ to be negative definite. This results in hidden genetic variance, reducing the additive genetic variance of all traits below the genic variance in $\mathbf{G}_g$ (Bulmer 1974; Lande 1980). When multiple traits are under selection, this so-called "Bulmer effect" also biases genetic correlations between traits. While each individual between-locus covariance $\mathbf{G}_b$ is very small when loci are unlinked, these covariances are $n-1$ times more numerous than within-locus covariance matrices $\mathbf{G}_w$, so the contribution of $\mathbf{G}_{LD}$ to $\mathbf{G}$ can become non-negligible when n is large (Walsh and Lynch 2018).

The magnitude of this effect can be quantified by

$$\mathbf{R} = \mathbf{G}^{-1/2}\mathbf{G}_{LD}\mathbf{G}^{-1/2} = \mathbf{G}^{-1/2}(\mathbf{G} - \mathbf{G}_g)\mathbf{G}^{-1/2} \tag{S13}$$

which represents a multivariate equivalent to the proportional reduction of genetic variance caused by hiding in linkage disequilibrium.

From (Lande 1980, eq. 17), the equilibrium between-locus additive genetic covariance matrix for equivalent and unlinked loci is $\mathbf{G}_b = -\frac{2}{(2n)^2}\mathbf{G}\mathbf{V}_s^{-1}\mathbf{G} = -\frac{1}{2n^2}\mathbf{G}\mathbf{V}_s^{-1}\mathbf{G}$, leading to

$$\mathbf{R} = -\frac{n-1}{2n}\mathbf{G}^{1/2}\mathbf{V}_s^{-1}\mathbf{G}^{1/2} \approx -\frac{n-1}{2V_s}\mathbf{G}_w^{1/2}\mathbf{V}_\rho^{-1}\mathbf{G}_w^{1/2}. \tag{S14}$$

The minus sign indicates that this effect overall reduces genetic variance, as expected. For a given $\mathbf{G}_w$ (approximately determined by the mutation-selection-drift balance derived above), the magnitude of this reduction increases with the number of loci n, and with strength of selection V_s^{-1}. We thus expect our analytical predictions for $\mathbf{G}$ and ρ_G to become less accurate under strong selection and with many loci, because of the increasing influence of stabilizing selection on linkage disequilibrium. Our simulations show that this is indeed the case: as the number of loci increases, the proportion of hidden genetic variance increases as predicted by equation (S14) (Fig. S6), and the influence of the strength of selection V_s^{-1} on genetic correlations is reduced (Fig. S7, Fig. S8).

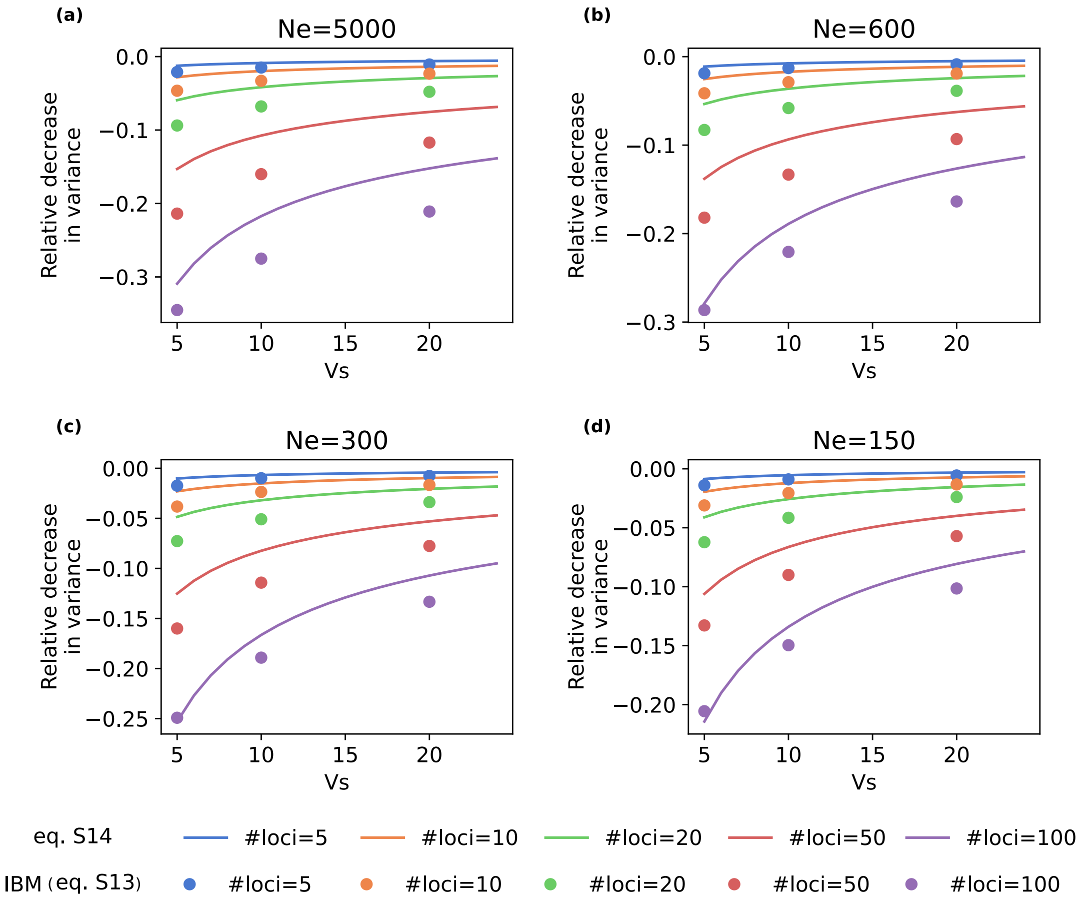

Figure S6: **Influence of the number of loci on hidden genetic variance.** (a-d) The relative decrease in genetic variance caused by linkage disequilibrium between loci, measured as the mean of the diagonal elements of matrix $\mathbf{R}$, is plotted against the width of the fitness peak V_s, for different effective sizes N_e. Points correspond to simulation results of individual-based model (IBM), where $\mathbf{R}$ was estimated as in equation S13 (rightmost member), with $\mathbf{G}_g$ computed as the sum of locus-specific covariance matrices $\mathbf{G}_{ii}$. Lines represents the analytical expectation obtained by replacing $\mathbf{G}$ in equation (S14) by its expectation at mutation-selection-drift equilibrium without linkage disequilibrium (eq. (9)). The parameter values for selection and mutation, corresponding to the Gaussian mutation regime, are the same as in Figure 1.

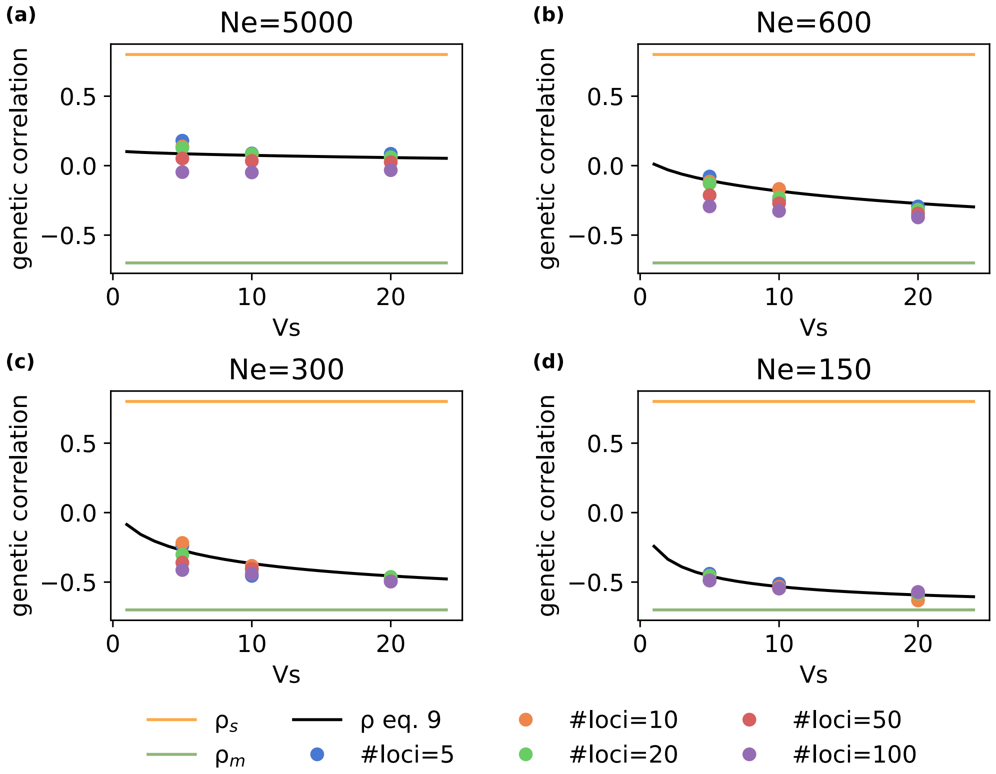

Figure S7: **Influence of the number of loci on genetic correlations at mutation-selection-drift equilibrium.** (a-d) The genetic correlation is plotted against the width of the fitness peak V_s, for different effective sizes N_e. The parameters values for selection and mutation, corresponding to the Gaussian mutation regime, are the same as in Figure 1. The number of loci ranges from 5 to 100. Note that as the number of loci becomes larger, genetic correlations change less with V_s.

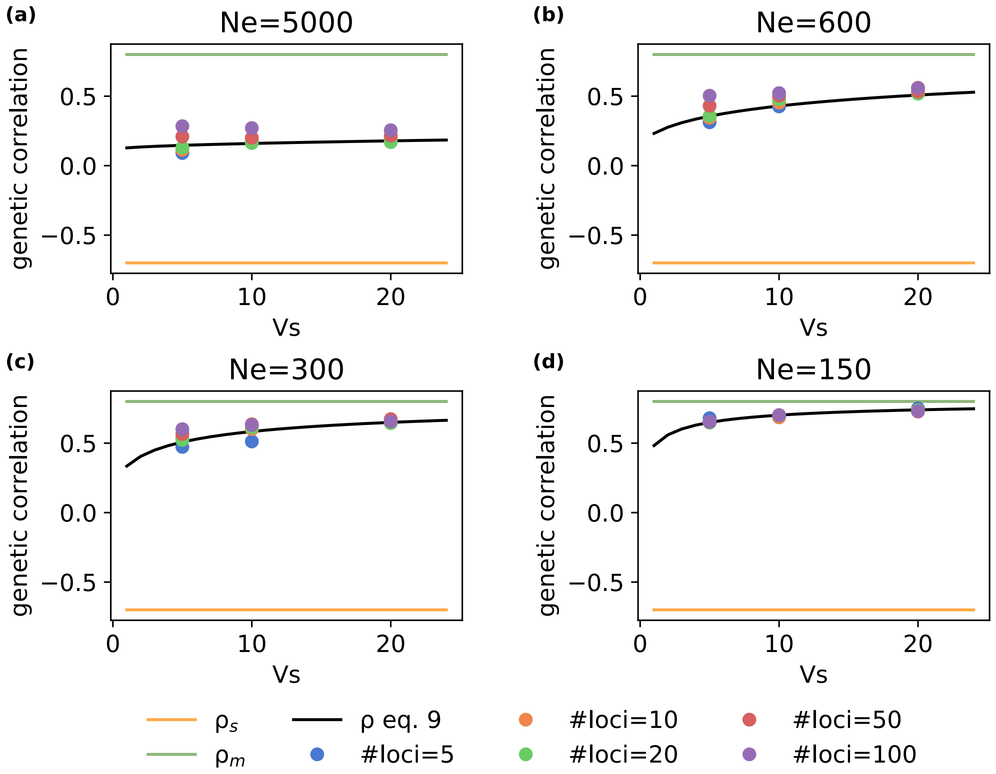

Figure S8: **Influence of the number of loci on genetic correlations at mutation-selection-drift equilibrium (Gaussian regimes, permuted correlations).** All parameters are the same as in Figure S7, but with permuted correlations, $\rho_m = 0.8$ and $\rho_s = -0.7$. This illustrates that a larger number of loci causes ρ_g to change towards ρ_m, rather than become smaller.

References

Bulmer, M.G. (1974). Linkage disequilibrium and genetic variability. *Genet. Res. (Camb.)* 23:281–289.

Bulmer, M.G. (1989). Maintenance of genetic variability by mutation - selection balance: A child's guide through the jungle. *Genome* 31:761–767.

Burger, R., Wagner, G.P. & Stettinger, F. (1989). How Much Heritable Variation Can be Maintained in Finite Populations by Mutation-Selection Balance? *Evolution* 43:1748.

Crow, J.F. & Kimura, M. (1970). An Introduction to Population Genetics Theory. Harper international edition.

Jones, A.G., Arnold, S.J. & Bürger, R. (2003). Stability of the G-matrix in a population experiencing pleiotropic mutation, stabilizing selection, and genetic drift. *Evolution* 57:1747–1760.

Kimura, M. (1965). A stochastic model concerning the maintenance of genetic variability in quantitative characters. *Proc. Natl. Acad. Sci. U.S.A.* 54:731–736.

Lande, R. (1979). Quantitative genetic analysis of multivariate evolution, applied to brain: body size allometry. *Evolution* 33:402–416.

Lande, R. (1980). The Genetic Covariance between Characters Maintained by Pleiotropic Mutations. *Genetics* 94:203–15.

Robertson, A. (1961). Inbreeding in artificial selection programmes. *Genet. Res. (Camb.)* 2:189–194.

Santiago, E. & Caballero, A. (1995). Effective size of populations under selection. *Genetics* 139.

Walsh, B. & Lynch, M. (2018). Evolution and Selection of Quantitative Traits. Oxford University Press.

Wright, S. (1969). Evolution and the Genetics of Populations, Volume 2: Theory of Gene Frequencies. University of Chicago Press.
